# Factors associated with HIV serodiscordance among couples in Mozambique: Comparison of the 2009 INSIDA and 2015 IMASIDA surveys

**DOI:** 10.1371/journal.pone.0234723

**Published:** 2020-06-16

**Authors:** Adelino J. C. Juga, Niel Hens, Nafissa Osman, Marc Aerts

**Affiliations:** 1 Department of Mathematics and Informatics, Faculty of Sciences, Eduardo Mondlane University, Maputo, Mozambique; 2 I-BioStat, Data Science Institute, Hasselt University, Diepenbeek, Belgium; 3 Centre for Health Economics Research and Modelling Infectious Diseases, Vaccine and Infectious Disease Institute (VAXINFECTIO), University of Antwerp, Antwerp, Belgium; 4 Epidemiology and Social Medicine (ESOC), Faculty of Medicine and Health Sciences, University of Antwerp, Antwerp, Belgium; 5 Department of Obstetrics and Gynecology, Maputo Central Hospital, Maputo, Mozambique; 6 Faculty of Medicine, Eduardo Mondlane University, Maputo, Mozambique; The Chinese University of Hong Kong, HONG KONG

## Abstract

Recent studies suggest that a large proportion of new HIV-1 infections in mature epidemics occurs within discordant couples, making discordancy a major contributor to the spread of HIV/AIDS in Africa. This paper aims at assessing changes over a five-year period (2009–2015) on the (risk) factors associated with HIV serodiscordance among couples in Mozambique, using cross-sectional data from the INSIDA and IMASIDA surveys. The pooled data of both surveys were analyzed using a joint model for three parameters characterizing in a particular way disagreement and sero(con/dis)corance between the HIV statuses of couples, as introduced by Aerts et al.: the probability that the female partner is HIV positive, given that both partners differ in their HIV status, the probability that only one partner is HIV positive, given that at least one of the two partners is positive (“positive” serodiscordance), and the probability that both partners are negative given that at most one of the two partners is positive (“negative” seroconcordance). The results reveal similar significant factors and estimates as in Aerts et al. (HIV prevalence, union number for woman, STI for man, condom use by woman and wealth index), but the additional significant factors “condom use by man” (no use had a negative effect on the positive serodiscordance) and “union number for man” (for couples where the man has been married or co-habiting with a woman before had a decreased negative seroconcordance) were identified. The only factor that had a different effect over time (IMASIDA as compared to INSIDA) was the effect of “HIV prevalence of province” on the negative seroconcordance. The negative effect of a higher HIV prevalence was less pronounced in 2015 for negative seroconcordance.

## Introduction

HIV continues to be a major global public health issue, having claimed more than 35 million lives so far. In 2017, 940 000 people died from HIV-related causes globally. There were approximately 36.9 million people living with HIV at the end of 2017 with 1.8 million people in 2017 globally. According to the World Health Organization (WHO), the African region is the most affected region, with 25.7 million people living with HIV in 2017. The African region also accounts for almost two-thirds of the global total of new HIV infections [1].

A large proportion of new HIV infections in mature epidemics occur within discordant couples, making discordancy a major contributor to the spread of HIV/AIDS in Africa [2,3]. Most of these new infections occurred within heterosexual and serodiscordant stable couple relationships [4]. In East Africa, 40% to 50% of the married or cohabitating HIV infected persons are in an HIV-discordant partnership [5]. Around 15% of Mozambican cohabiting couples were infected by HIV in 2009; that is, either one or both members are HIV-positive [3]. This rate increased to 16,7% in 2015 [6], suggesting that HIV discordance (when one member of a couple is HIV-positive and the other is HIV negative) may be responsible for a substantial percentage of all new HIV infections [7]. Global estimates of HIV serodiscordance vary from country to country (e.g. in Vietnam it was 58% in 2016 [8]). In Mozambique, the global estimate of HIV serodiscordance was 0.68 (95%CI [0.63, 0.72]) in 2009 [9], which decreased to 0.57(95%CI [0.52, 0.62]) in 2015.

Risk factors associated with HIV serodiscordance among couples have been widely investigated [4, 10, 11, 9, 12]. Eyawo et al. [4] reviewed several published as well as unpublished articles and found that women were as likely as men to be the index partner in discordant couple. A case-control study conducted by Habte et al. [11] in Ethiopia found that 5.8% and 2.6% of all couples were either male and female discordant, respectively. This study reveals that rare use of condom and active tuberculosis at enrolment were predictors associated with serodiscordance. On the other hand, Uah et al. [10] found that the probability of a couple being female discordant was associated with a history of at least two terminations of pregnancy and five or more total lifetime sexual partnerships. The HIV status disclosure rate in this study was 83.7%; with significantly higher disclosure rate in concordant couples (83.3%) compared to 76.8% among women in serodiscordant relationships.

Using the second Mozambican demographic health survey (DHS) data conducted in 2009, designated as INSIDA, Fishel et al. [3] conducted the first serodiscordance analysis. Their research used two separate logistic regression models. One model compared concordant positive couples with all discordant couples while the second model compared concordant positive couples with male discordant as well as with female discordant couples [3]. Their study did find only a weak association between HIV serodiscordance and polygamy status (yes/no) as well as between HIV serodiscordance and the amount of time passed since the couple last had sexual intercourse [3]. Juga et al. [9] and Aerts et al. [13] re-analyzed the INSIDA data by fitting flexible marginal and random effects models to adequately and directly describe the conditional “positive” serodiscordance measure (CDM, defined as the probability that only one partner is HIV positive, given that at least one of the two partners is positive), as a new alternative discordance measure of the HIV status for woman and man within a couple. Aerts et al. [13] found that the HIV prevalence for the province where a couple was located (varying from 3,7% in Niassa to 25.1% in Gaza [6]), the “union number for woman”, the “STI of man” (presence of symptoms of sexually transmitted infections), and the “wealth index” were associated with the serodiscordance measure CDM. In addition, the probability to be serodiscordant did vary across enumeration areas (EAs).

Mozambique conducted the third DHS survey in 2015, designated as the IMASIDA survey. In this paper, we use the model of Aerts et al. [13] on the pooled dataset of the INSIDA and IMASIDA surveys. while accounting for the study designs of both surveys. Main objective is to investigate the hypothesis that there are no changes over the five-year period (2009–2015), based on both surveys; and more specifically the hypothesis that the same risk factors have the same effect on the CDM. Additionally, it is of interest whether this hypothesis of no changes also holds for the other two parameters in the model: the probability that the female partner is HIV positive, given that both partners differ in their HIV status, and the probability that both partners are negative given that at most one of the two partners is positive.

## Materials and methods

### INSIDA and IMASIDA study design

The first Mozambican national and demographic health survey of prevalence, risk behavioural and information about HIV and AIDS (INSIDA) was conducted in 2009 [3] while the second, designated as the IMASIDA survey [6], took place in 2015. These two surveys were designed to collect comprehensive data on the prevalence of HIV infection, knowledge, attitude, behaviour risk factors and access to information on HIV and AIDS in the Mozambican population. Both surveys were cross-sectional two-stage surveys. Moreover, stratification and cluster sampling methods were applied to ensure that for each province inference was possible with nearly the same precision. EAs, households and individuals were Primary Sampling Units (PSU), Secondary Sampling Units (SSU) and Tertiary Sampling Units (TSU) respectively [3, 6].

The INSIDA survey selected randomly 270 EAs from 45000 EAs defined according to the 2007 general population and housing census. A total of 122 EAs were sampled from urban areas and 148 EAs from rural areas. In the IMASIDA survey, 307 EAs were also randomly sampled from 45000 EAs (from 2007 general population and housing census), 134 EAs from in urban areas and 173 EAs from rural areas. A fixed number of households were systematically selected within each EA in both surveys [3, 6]. Men and women aged between 15–59 years were eligible to participate in an individual interview and to provide a blood sample for HIV testing. During the individual interviews, respondents were asked if they were married, and if so, who their spouse/wife was. If the spouse/wife was named in the household questionnaire, the interviewer recorded the household line number of the spouse/wife in the individual’s questionnaire. Through a methodology standardized by the DHS project, cohabiting couples are matched together, allowing for an analysis of their HIV status and other characteristics. The datasets of both surveys are accessible at https://dhsprogram.com/data/available-datasets.cfm by selecting Mozambique, the year of the survey, and submitting a research project proposal.

### Description of variables

Variables used in the analysis are briefly described in Table 1. The HIV status (infected yes/no) for both members within a couple are the (binary) response variables of interest. The following covariates were used in the model building process. The variable wealth index refers to the economic status of the couple and condom use by man and woman refers to whether the male, respectively female partner used a male, respectively female condom the last time the man, respectively woman had sexual intercourse with the other partner. Union number for woman/man refers to whether the individual has been married or cohabiting with a man/woman once or more than once. Genital discharge refers to whether a woman had genital discharge while genital sore/ulcer refers to whether a woman had a genital sore/ulcer in the last 12 months. The number of partners for woman and man refers to the total number of sexual partners including the current partner in the last 12 months.

**Table 1:** INSIDA and IMASIDA variables: Basic description of variables.

Variables name	Type	Categories
HIV status for woman	Binary	0: HIV Negative (ref. category)
		1: HIV Positive
HIV status for man	Binary	0: HIV Negative (ref. category)
		1: HIV Positive
Union number for woman	Binary	0: once (ref. category)
		1: more than once
Union number for man	Binary	0: once (ref. category)
		1: more than once
Genital discharge for woman	Binary	0: no (ref. category)
		1: yes
Genital sore/ulcer for woman	Binary	0: no (ref. category)
		1: yes
Condom use by man (male condom)	Binary	0: no (ref. category)
		1: yes
Condom use by woman (female condom)	Binary	0: no (ref. category)
		1: yes
Survey indicator	Binary	0: INSIDA (ref. survey)
		1: IMASIDA
Male circumcision	Binary	0: no (ref. category)
		1: yes
STID for man	Binary	0: no (ref. category)
		1: yes
Couple’s wealth index	Multicategory	0: poor
		1: middle
		2: rich (ref. category)
HIV Prevalence of province	Multicategory	0: [< 5%] (ref. category)
		1: [5%–15%]
		2: [> 15%]
Number of partners for woman	Discrete	-
Number of partners for man	Discrete	-

The survey indicator is a binary variable indicating whether it concerns an observation of the INSIDA or the IMASIDA survey. The HIV prevalence for province was categorized into three categories using cut points of 5% and 15%. Male circumcision shows whether the male partner was circumcised or not. STID refers to whether the man had sexually transmitted infectious disease (STID) or symptoms of STID in the past 12 months. The variable Condom use by man had 1.24% of missingness, followed by Genital discharge and Genital sore/ulcer with 0.74% and Union number for man with 0.49% of missing values. However, analyses of this paper were performed using complete cases only.

### Measuring con(dis)cordance

Suppose that *y*_*ij*_ = (*y*_*ij*1_, *y*_*ij*2_) denotes the HIV status (1 if positive, 0 if negative) of a (female, male)-couple *j* = 1, …, *n*_*i*_ in EA *i* with *n*_*i*_ sampled couples, *i* = 1, …, *N* and *x*_*ij*_ vectors of covariates or risk factors. For the INSIDA data, a total of $\sum_{i=1}^{N}n_{i}=2159$ couples were sampled while a total of $\sum_{i=1}^{N}n_{i}=2021$ couples were sampled in the IMASIDA survey. A relationship between the outcome vectors *y*_*ij*_ and the covariates vectors *x*_*ij*_ can be established. In the sequel, the dependency of *x*_*ij*_ on the indices *i* and *j* as well as the dependency of parameters on *x*_*ij*_ is sometimes suppressed to simplify notation.

The model arises from the decomposition of the joint probabilities (given covariate variables *x*_*ij*_)

$$\pi_{{\text{j}}_{1},\;{\text{j}}_{2}}(x_{ij})=\text{P}({\text{y}}_{\text{ij}1}={\text{j}}_{1},{\text{y}}_{\text{ij}2}={\text{j}}_{2}\;|x_{ij}\;),\;{\text{j}}_{1},\;{\text{j}}_{2}=0,\;1$$ (1)

with ${0<\pi }_{{\text{j}}_{1},\;{\text{j}}_{2}}(x_{ij})<1$ and $\sum_{j_{1},j_{2}=0}^{1}\pi_{{\text{j}}_{1},\;{\text{j}}_{2}}(x_{ij})=1\;$. Typically these joint probabilities are reparametrized in terms of *π*_1_+(*x*_*ij*_) = *π*_10_(*x*_*ij*_) + *π*_11_(*x*_*ij*_), and *π*_+1_+(*x*_*ij*_) = *π*_01_(*x*_*ij*_) + *π*_11_(*x*_*ij*_), the “marginal” probability of a positive HIV test for the female and male partner respectively, together with an “association” parameter *θ*(*x*_*ij*_), relating the HIV status of both partners. Statistical models then specify the functional dependency of the parameters *π*_1+_(*x*_*ij*_), *π*_+1_(*x*_*ij*_) and *θ*(*x*_*ij*_) on the covariates and risk factors *x*_*ij*_. In literature, there are many dependency parameters such as the correlation, $\rho =(\pi_{11}-\pi_{1+}\pi_{+1})/\sqrt{\pi_{1+}(1-\pi_{1+})\pi_{+1}(1-\pi_{+1})}$, the odds ratio (OR), *ϕ* = (*π*_00_*π*_11_)/(*π*_01_*π*_10_), and the dependence ratio (DR), *τ* = *π*_11_/(*π*_01_*π*_10_). Faes et al. [14] stated that although the OR is an attractive association measure with nice mathematical properties, it is less suitable to quantify concordance due to its symmetry, treating 0±0 matches of equal importance as 1±1 matches. Following the same ideas as Faes et al. [14], Juga et al. [9] defined the HIV conditional “positive” (sero)discordance measure (CDM) as

$$\text{CDM}=P({\text{y}}_{\text{ij}1}\neq {\text{y}}_{\text{ij}2}|{\text{y}}_{\text{ij}1}+{\text{y}}_{\text{ij}2}\geq 1)=\frac{\pi_{10}+\pi_{01}}{\pi_{10}+\pi_{01}+\pi_{11}},$$

the conditional probability that the couple is HIV discordant, given that at least one of them, man or woman, is HIV positive, They showed that the CDM measure is a more direct and relevant measure to study the effects of risk factors associated with HIV serodiscordance.

Aerts et al. [13] proposed a new improved parameterization based on three parammeters: the CDM as defined above, the conditional “negative” (sero)concordance measure (CSM) being the probability that both are agreeing (both negative), given that at most one is positive:

$$\text{CSM}=P({\text{y}}_{\text{ij}1}={\text{y}}_{\text{ij}2}|{\text{y}}_{\text{ij}1}+{\text{y}}_{\text{ij}2}\leq 1)=\frac{\pi_{00}}{\pi_{00}+\pi_{10}+\pi_{01}},$$

and the conditional probability that the female partner is positive, given that both disagree:

$$\text{CFP}=P({\text{y}}_{\text{ij}1}=1|{\text{y}}_{\text{ij}1}\neq {\text{y}}_{\text{ij}2})=\frac{\pi_{10}}{\pi_{10}+\pi_{01}}.$$

For more details on these parameters, their properties and their joint estimation, see Aerts et al. [13].

### Joint marginal and random effect model specification

The dependency of the three conditional probabilities CDM, CSM and CFP on covariates can be modeled with three components:

$${\text{h}}_{1}(\text{CFP}(x_{ij}))=\beta_{1}^{T}x_{ij},\;{\text{h}}_{2}(\text{CDM}\;(x_{ij}))=\beta_{2}^{T}x_{ij},\;{\text{h}}_{3}(\text{CSM}(x_{ij}))=\beta_{3}^{T}x_{ij},$$

where h_1_, h_2_, and h_3_ are appropriate link functions. We prefer the logit function as in classical logistic regression model, since all parameters are probabilities, and the logit link allows the interpretation of covariate effects in terms of odds ratios. The parameters $\beta_{1}^{T},\beta_{2}^{T},\;\beta_{3}^{T}$ are unknown regression coefficients (intercept and slopes) for each the set of covariates *x*_*ij*_ and can be different or (partly) in common.

As CDM, CSM and CFP are expected to be heterogeneous across EAs and should reflect the design of the surveys, the logit models have to be extended with an EA random effect as follows, expressing that the covariates x_1,ij_, x_2,ij_ and x_3,ij_ can be possibly different subvectors of the full covariate/factor x_ij_:

$$\begin{array}{l} \text{logit}\left(\text{CFP}\left(x_{1,ij}\right)\right)=\beta_{1}^{T}x_{1,ij}+b_{\text{CFP},i},\;\\ \text{logit}\left(\text{CDM}\;\left(x_{2,ij}\right)\right)=\beta_{2}^{\text{T}}{\text{x}}_{2,\text{ij}}+{\text{b}}_{\text{CDM},\text{i}},\\ \text{logit}\left(\text{CSM}\left(x_{3,ij}\right)\right)=\beta_{3}^{T}x_{3,ij}+b_{\text{CSM},i} \end{array}$$ (2)

where

$$({\text{b}}_{\text{CFP},\text{i},}\;{\text{b}}_{\text{CDM},\text{i},}\;{\text{b}}_{\text{CSM},\text{i}})\sim {\text{N}}_{3}(0,\sum ),$$

are trivariate normally distributed random EA-effects, with mean zero-vector and covariance matrix ∑. The variance components $\sigma_{\text{CFP}}^{2},\;\sigma_{\text{CDM}}^{2}$ and $\sigma_{\text{CSM}}^{2}$ (diagonal elements of the ∑-matrix) quantify the degree of heterogeneity across the AEs for each of the three parameters. Different choices for the covariance matrix were considered (partial and full-correlated random effects; partial and full shared random effects; partial and full equal random effects and lastly independent random effects). For more details on the different models and covariance matrix ∑ specifications and the estimation of all parameters with maximum likelihood, we refer to Aerts et al. [13].

#### Accounting for survey design

Both INSIDA and IMASIDA surveys used stratification and clustering sampling methods, with a two-stage sampling procedure to access individuals within a household. This type of survey may sample certain groups or strata at higher rates than others, in order to ensure sufficient precision for group-specific estimates and for bias reduction. We assigned weights to observations to account for this over-sampling. For more details on the INSIDA and IMASIDA weights calculation, we refer to Juga et al. [9] and Reed [6], respectively.

## Results

Table 2 shows the number of parameters, the Akaike information criterion (AIC, smaller values indicate a better fit [15]) and Bayesian information criterion (BIC, smaller values indicate a better fit, BIC tends to select simpler models than AIC [16]) goodness of fit measures of all fitted models. The last two columns show the models' ranks according to their AIC and BIC values.

**Table 2:** Comparison of marginal model, and full-shared, partial-shared, full-equal partial-equal, full-independent, partial-correlated and full-correlated random-effects models.

Model	Number of parameters	AIC	BIC	Rank according to AIC	Rank According to BIC
MM (marginal model)	22	4893.0	5030.9	8	8
PS (partial-shared RE model)	24	4838.2	4927.6	2	2
FS (full-shared RE model)	25	4840.2	4933.3	4	4
PE (partial-equal RE model)	23	4841.2	4926.9	5	1
FS (full-equal RE model)	23	4852.5	4938.2	7	6
FS (full-independent RE model)	24	4846.1	4935.5	6	5
**PC (partial-correlated RE model)**	**25**	**4836.4**	**4929.4**	**1**	3
FC (full-correlated RE model)	28	4838.3	4942.6	3	7

According to AIC, the model with partial-correlated random effects (*b*_CDM,*i*_, *b*_CSM,*i*_) and no random effect (*b*_CFP,*i*_) is the best fitting model, closely followed by a partial-shared random-effects model (AIC values of 4836.4 and 4838.2 respectively). The best model according to BIC is the partial-equal random-effects model, closely followed by the partial-shared random-effects model and the partial-correlated random effects model. We opted for the partial-correlated random effects as our final model, as the same model was selected by Aerts et al. [13], facilitating the comparison of the new analysis on the pooled data with the analysis on the INSIDA survey only. At first glance, the new extended analysis is very much in line with that of Aerts et al. [13]. The factors “HIV prevalence of province”, “union number for woman”, “STID for man” and “wealth index” are significant for one or more parameters CFP, CDM and CSM and the estimates are quite similar. On the other hand, “condom use by woman” which was having a significant effect on the CFP, is no longer significant for any of the parameters. Some other additional significant factors showed up: “condom use by man” (for CDM) and “number of partners of man” (for CFP). The random effect of enumeration was significant for both parameters CDM and CSM, with variance components estimates smaller but more precise as those obtained by Aerts et al. [13]. The correlation between those random effects was negative (-0.29 with se = 0.11), indicating that, while correcting for the other factors and across EAs, couples with a higher value for CDM tend to have a lower value for CSM.

Our analysis shows that “union number for woman” and “number of partners of man” have a significant effect on the CFP. The woman having been married or cohabitated with a man more than once, and the man having more sexual partners in the last 12 months, increases the probability that the female partner is HIV positive, given that both partners differ in their HIV status (ORs 1.84 and 1.21 respectively). For the CDM, all factors “HIV prevalence of province”, “condom use by man” and “STID of man” have a significant negative effect. A province with a higher prevalence of HIV, a couple of which the man did not use a condom the last time he had sexual intercourse with his partner, and a couple of which the man had a sexually transmitted infectious disease or symptoms thereof in the past 12 months, corresponds to a lower probability that only one is HIV positive, given that at least one of the two partners is positive (ORs {0.37,0.32}, 0.53 and 0.21 respectively).

Next, our analysis shows also a negative effect of “HIV prevalence of province”, “union number for woman”, “union number for man” and the “survey indicator” on the CSM. The man and/or woman having been married or cohabited with a partner before decreases the probability that both are negative given that at most one of the two partners is positive (OR = 0.54 for woman, OR = 0.78 for man, OR = 0.42 for both). The survey indicator only appears in the model for the CSM, with a main effect and an interaction term with “HIV prevalence of province”. A province with a higher prevalence of HIV implies a lower probability that both partners are negative given that at most one of the two partners is positive (ORs {0.45,0.23} for the INSIDA survey and {exp(-0.79+0.49) = 0.74, exp(-1.46+1.01) = 0.64} for the IMASIDA survey). So, the effect of the “HIV prevalence of province” is weaker in the IMASIDA. Moreover, regardless of the level of prevalence of HIV, and controlling for all factors in the model, the CSM is significantly lower in the second survey. Finally, the “wealth index” has a positive effect, implying that being a “poorer” or “middle” category couple has a higher probability that both partners are negative given that at most one of the two partners is positive (OR = 1.97 and 1.63 and respectively), again while correcting for the effects of all other factors (Table 3).

**Table 3:** Estimates (standard errors) of the final model with partial-correlated random effects (*b*_CDM,*i*_, *b*_CSM,*i*_) with and no random effect (*b*_CFP,*i*_).

Effects	CFP	CDM	CSM
**Intercept**	-0.76(0.20)*	1.51(0.30)*	3.10(0.24)*
**HIV prevalence of province**			
[5–15%]	-	-0.99(0.28)*	-0.79(0.24)*
[> 15%]	-	-1.15(0.30)*	-1.46(0.26)*
**Wealth index**			
Poorer	-	0.16(0.22)	0.68(0.15)*
Middle	-	0.39(0.25)	0.49(0.16)*
**Condom use by woman**			
No	0.79(0.45)	-	-
**Condom use by man**			
No	-	-0.64(0.22)*	-
**STID for man**			
Yes	-	-1.55(0.23)*	-
**Union number for woman**			
More than once	0.61(0.24)*	-0.33(0.18)	-0.62(0.13)*
**Union number for man**			
More than once	-	-	-0.25(0.10)*
**# of partners for man**	0.19(0.09)*	-	-
**Survey indicator**			
IMASIDA	-	-	-0.73(0.25)*
**Survey indicator x HIV prevalence of province**			
IMASIDA x [5–15%]	-	-	0.49(0.30)
IMASIDA x [> 15%]	-	-	1.01(0.32)*
**Variance Components**			
*σ*^2^_CDM_	-	0.36(0.16)†	
*ρ*_CDM,CSM_	-	-0.29(0.11)*	
*σ*^2^_CSM_	-	0.47(0.11)†	

*Significant at 5% level based on a likelihood ratio test,

^†^ Significant at 5% level, using $\chi_{0,\;1}^{2}$ mixture.

## Conclusions and discussion

This paper investigated the relationship/association between the HIV status of woman and man within a couple and the risk factors in terms of three conditional probabilities, as introduced by Aerts et al. [13]: the CFP (being the probability that the female partner is HIV positive, given that both partners disagree in their HIV status), the CDM that only one partner is HIV positive, given that at least one of the two partners is positive (“positive” serodiscordance), and the probability CSM that both are negative given that at most one of the two partners is positive (“negative” seroconcordance).

The new extended analysis based on the INSIDA(2009) and the IMASIDA(2015) survey reveals similar significant factors and estimates as in the more limited analysis of Aerts et al. [13] on the INSIDA survey only. But the additional significant factors “condom use by man” (no use had a negative effect on the CDM) and “union number for man” (for couples where the man has been married or cohabited with a woman before had a decreased CSM) were identified. The only factor that had a different effect over time (IMASIDA as compared to INSIDA) was the effect of “HIV prevalence of province” on the CSM. The negative effect of a higher HIV prevalence was less pronounced in 2015 for the CSM.

The results found in this paper are in line with the results found in other studies, although applying different methodologies [9,13]. A Kenyan study among persons aged 18 months to 64 years, found that factors associated with HIV-discordance were “an increasing number of lifetime sexual partners for women” and “lack of male circumcision” and “reporting sexual partners outside of the relationship in the past 12 months for men”. This study underscored the importance of couple-based prevention strategies that focus on reducing sexual transmission of HIV among couples including voluntary medical male circumcision, reduction of number of sexual partners, routine couples HIV counseling and testing and knowledge and disclosure of partner status [17].

A cross-sectional study conducted in Ethiopia with 154 couples, indicated a high prevalence of HIV discordance and increased risk of vulnerability. They recommended that couples should be aware of their own and their partners’ serostatus before and after engagement. Furthermore, the following risk reduction methods were recommended: education of discordant couples on 100% correct condom use, and if condom breaks, or if they forget to use, Post-Exposure Prophylaxis must be established; for those who did not volunteer to use condom and/or have a child, early initiation of ART to positive partner [18].

Based on our findings, we recommend the Mozambican health authorities to promote HIV testing for women and men prior to marriage or living together as well as regular testing for couples. These programs would support couples to prevent transmission once one partner has become infected. Juga et al. [9] and Kaiser et al. [17] also advocated that prevention interventions should begin early in relationships and include mutual knowledge of HIV status.

Finally, our study has some important limitations. Both surveys are cross-sectional. Some couples might have participated in both studies, but as no couple ID was available, our model and analysis could not reflect such double participation. Nevertheless, given the design of the studies and the random selection of couples, it is to be expected that the large majority of couples in both surveys is different. A design in which the same couples were included in the second survey (matched couples) would facilitate a study of which factors have an effect on a change in the positive serodiscordance and negative seroconcordance status within a couple.

Another limitation of the surveys and our study is that couples, who were unmarried or not cohabiting together, such as teenagers and young adults, were not included. The surveys only provide data of cohabiting couples who, during the individual interviews, responded to be married or living together, and who identified their spouse/wife. Partner concurrency is also a common finding in serodiscordant couples. In both surveys INSIDA and IMASIDA, a particular question was informing about other sexual partners besides the partner of the couple. However, missingness of this variable was of the order of 60%. As one cannot assume such missingness to be ignorable and as methodology for non-ignorable missingness is complicating analysis considerably, we decided to not include this variable.

## Supporting information

S1 Appendix SAS code for the final partial-correlated random EA-effects model (see results in Table 3). (DOCX)

S1 Data (XLSX)
