# Supplementary material for: Factors associated with HIV serodiscordance among couples in Mozambique: Comparison of the 2009 INSIDA and 2015 IMASIDA surveys
Source: PLoS One. 2020 Jun 16;15(6):e0234723. doi: 10.1371/journal.pone.0234723 (PMC7297311; doi:10.1371/journal.pone.0234723)
Supplement: S1 Appendix — (DOCX) [file pone.0234723.s001.docx]

**Appendix: SAS code for the final partial-correlated random EA-effects model (see results in Table 3)**

**PROC** **NLMIXED** DATA=INSIDA_IMASIDA1 QPOINTS=**3**;

/* CFP=P(1,0)/[P(0,1)+P(1,0)]*/
ETA1 = B0_1 + B1_1*TOT_LIFETIME_NUMB_PART_W + B2_1*N_UNION_DW2 + B3_1*CONDOM_DW1;
CFP=**1**/(**1** + EXP(-ETA1));

/* CDM) = [P(1,0)+P(0,1)]/[P(1,0)+P(0,1)+P(1,1)]*/
ETA2 = - (U2 + B0_2 + B1_2*PREV_D2 + B2_2*PREV_D3 + B3_2*PO + B4_2*MID + B5_2*CONDOM_DM1 + B6_2*STID_dM1 + B7_2*N_UNION_DW2);
CDM = **1**/(**1** + EXP(-ETA2))

/* CSM = P(0,0))/[P(0,1)+P(1,0)+P(0,0)]*/
ETA3 = U3 + B0_3 + B1_3*PREV_D2 + B2_3*PREV_D3 + B3_3*N_UNION_DW2 + B4_3*N_UNION_DM2 + B5_3*PO + B6_3*MID +B7_3*(IND_SURV=**1**) + B8_3*PREV_D2*(IND_SURV=**1**) + B9_3*PREV_D3*(IND_SURV=**1**);
CSM = **1**/(**1** + EXP(-ETA3));

P11 = (CDM-CSM*CDM)/(**1**-CSM*CDM);
P00 = (CSM-CSM*CDM)/(**1**-CSM*CDM);
P10 = CFP*(**1**-CSM)*(**1**-CDM)/(**1**-CSM*CDM);
P01 = (**1**-CFP)*(**1**-CSM)*(**1**-CDM)/(**1**-CSM*CDM);

/* COMPUTE LOG LIKELIHOOD FOR BIVARIATE OBSERVATIONS */
LL = HIV_W1*HIV_M1*LOG(P11) + HIV_W1*(**1**-HIV_M1)*LOG(P10) + (**1**-HIV_W1)*HIV_M1*LOG(P01) +(**1**-HIV_W1)*(**1**-HIV_M1)*LOG(P00);
LL = LL*WEIGHT; MODEL LL ~ GENERAL(LL);
RANDOM  U2 U3~NORMAL([**0**,**0**],[D11,D21,D22]) SUBJECT=EA;

**RUN**;
